# Socioeconomic and Demographic Determinants of Hip Fracture Incidence: A Comprehensive Analysis

**DOI:** 10.7759/cureus.68790

**Published:** 2024-09-06

**Authors:** Zeeshan Hussain, Abdullah Bin Sahl, Ahad Hussain, Tom Collins, Anand Pillai

**Affiliations:** 1 Trauma and Orthopaedics, University of Manchester, Manchester, GBR; 2 Trauma and Orthopaedics, Wythenshawe Hospital, Manchester University NHS Foundation Trust, Manchester, GBR; 3 General Practice, Carpenters Surgery, AT Medics, London, GBR

**Keywords:** demographic, epidemiology, fracture, geriatrics, hip fracture, index of multiple deprivation (imd), neck of femur, orthopaedics, socioeconomic

## Abstract

Introduction: Hip fracture incidence is rising globally, making it crucial to understand the demographic factors that influence their occurrence for targeted healthcare interventions. This study focuses on examining the determinants of hip fracture incidence, particularly socioeconomic status, body mass index (BMI), and gender.

Methods: The study utilised data from 570 patients recorded in the 2023 National Hip Fracture Database Audit tool. Socioeconomic status was assessed through the Index of Multiple Deprivation (IMD), based on patient postcodes. The analysis aimed to explore correlations between socioeconomic status, BMI, gender, and the incidence and types of hip fractures.

Results: The findings indicate that the majority of hip fractures occurred in individuals from the lowest socioeconomic deciles, with a statistically significant correlation (p < 0.05) between lower socioeconomic status and higher fracture incidence. Gender-specific disparities were observed, with males experiencing fractures at younger ages and presenting with different fracture types compared to females. Additionally, lower BMI was significantly associated with a higher likelihood of certain fracture types (p < 0.05).

Conclusions: The study highlights the substantial influence of socioeconomic status, gender, and BMI on hip fracture incidence and typology. These results indicate the need for targeted prevention strategies and health policies aimed at mitigating hip fracture risks in socioeconomically disadvantaged populations. Understanding these factors can enable healthcare providers to allocate resources and interventions more effectively, reducing the incidence and impact of hip fractures in deprived areas.

## Introduction

The UK witnessed a staggering 72,160 hip fractures in 2022 [1], marking a 6,000 patient increase compared to the previous two years (2021 and 2020) when hip fracture incidence remained below 66,000. A quarter of these patients will experience another hip fracture within five years, with over 50% of these recurrent fractures occurring within 18 months [1]. The incidence of hip fractures was estimated at 14.2 million globally in 2019 [2], with a one-year postoperative mortality rate of 28.3% [3].

Hence, the primary consequence of a hip fracture is increased mortality [4] alongside a significant decrease in health-related quality of life [5]. The healthcare costs associated with hip fracture patients are more than double those of patients without them [6], further burdening the healthcare system financially.

A 2015 study by Newton et al. [7] analysed data from the Global Burden of Disease Study 2013, examining trends in health inequalities across different regions and levels of deprivation in England. It highlighted persistent health gaps despite overall improvements in health outcomes. Another study [8] adapted the Indices of Multiple Deprivation (IMD) for use across the UK to analyse mortality rates, revealing significant inequalities in health outcomes based on deprivation levels. The studies in the literature are, however, conflicting on whether social deprivation affects hip fracture risk. A 2015 study found a 30% increased incidence of hip fractures in wealthier areas [9]. Conversely, other studies [10,11] have identified that individuals living in socioeconomically deprived areas are at an increased risk of experiencing hip fractures and often at a younger age [12] compared to their less deprived counterparts. These individuals also face poorer outcomes post-fracture, including longer hospital stays and a greater likelihood of readmission. An alternative study [9] indicated that increased deprivation did not correlate with early mortality risk but was associated with a 5.6-year earlier age of presentation, with deprived patients more likely to require further acute hospital admissions.

Research has shown that lower body mass index (BMI) is associated with an increased risk of hip and osteoporotic fractures with this relationship being independent of age and sex [13]. Although no studies specifically investigate the potential link between BMI and the type of hip fractures a patient may experience due to their BMI, it is well-documented that extreme BMI levels are linked to increased complications and mortality following hip fracture surgery [14]. The relationship between BMI and hip fracture risk also varies by sex. A 2018 study [15] demonstrated that sex and age significantly influence fracture risks associated with body weight.

Women generally exhibit a higher incidence of hip fractures compared to men, often attributed to factors such as lower bone mineral density and higher life expectancy [16]. Moreover, men typically have higher mortality rates and are more likely to experience severe complications post-fracture compared to women [17]. A comparative study [18] found that the proportion of intertrochanteric fractures increases with age in women, a pattern not observed in men, possibly due to differences in the type and rate of bone loss between genders.

This study aims to investigate the impact of socioeconomic status, BMI, and gender on the incidence and typology of hip fractures, with the goal of informing targeted healthcare strategies and resource allocations for high-risk populations.

## Materials and methods

Our study utilised data collected from the National Hip Fracture Database Audit tool [19] for patients who sustained hip fractures between the 1st of January 2022 and 31st of January 2023 and underwent surgical intervention at Wythenshawe Hospital (MFT-W). We included all patients who sustained a hip fracture in one or both hips and were admitted to our hospital and excluded the patients who did not have a postcode in the UK at the time of admission from this data. 

We aimed to investigate various demographic factors and their influence on the incidence and type of hip fractures, specifically focusing on age, BMI, gender, and the level of deprivation in the area of residence. Additionally, we sought to identify any demographic correlations and their links to patient care management, such as length of stay and patient mobility. 

In this study, the collected data were compiled into an anonymised dataset and subsequently imported into IBM SPSS Statistics for Windows, Version 29 (Released 2023; IBM Corp., Armonk, New York, United States) for comprehensive analysis. Chi-square tests were employed to assess the associations between various categorical variables, including socioeconomic status, BMI, gender, and the types of hip fractures. The significance of the results was determined by interpreting the p-values, with a threshold of p < 0.05 considered indicative of a statistically significant association. Where the p-value exceeded 0.05, the relationship between the variables was deemed not statistically significant.

We utilised the IMD from the English Indices of Deprivation database to categorise patients' socioeconomic status based on their residential postcodes. This database compiles deprivation data across seven key domains: income, employment, education, health, crime, barriers to housing and services, and the quality of the living environment. Each domain is weighted and combined to compute the IMD score for every lower-layer super output area (LSOA) in England, encompassing 32,844 LSOAs. These LSOAs are ranked from the most deprived (rank 1) to the least deprived (rank 32,844), with each decile containing 3,284 LSOAs. This is presented in Table 1.

**Table 1:** Index of Multiple Deprivation (IMD) deciles and lower-layer super output area (LSOA) categories

IMD decile	Lower layer super output areas (LSOAs)
1 (most deprived)	1-3,284
2	3,285 to 6,568
3	6,569 to 9,583
4	9,584 to 13,137
5	13,138 to 16,422
6	16,423 to 19,706
7	19,707 to 22.990
8	22,991 to 26,275
9	26,276 to 29,559
10	29,560 to 32,844

**Figure 1:**
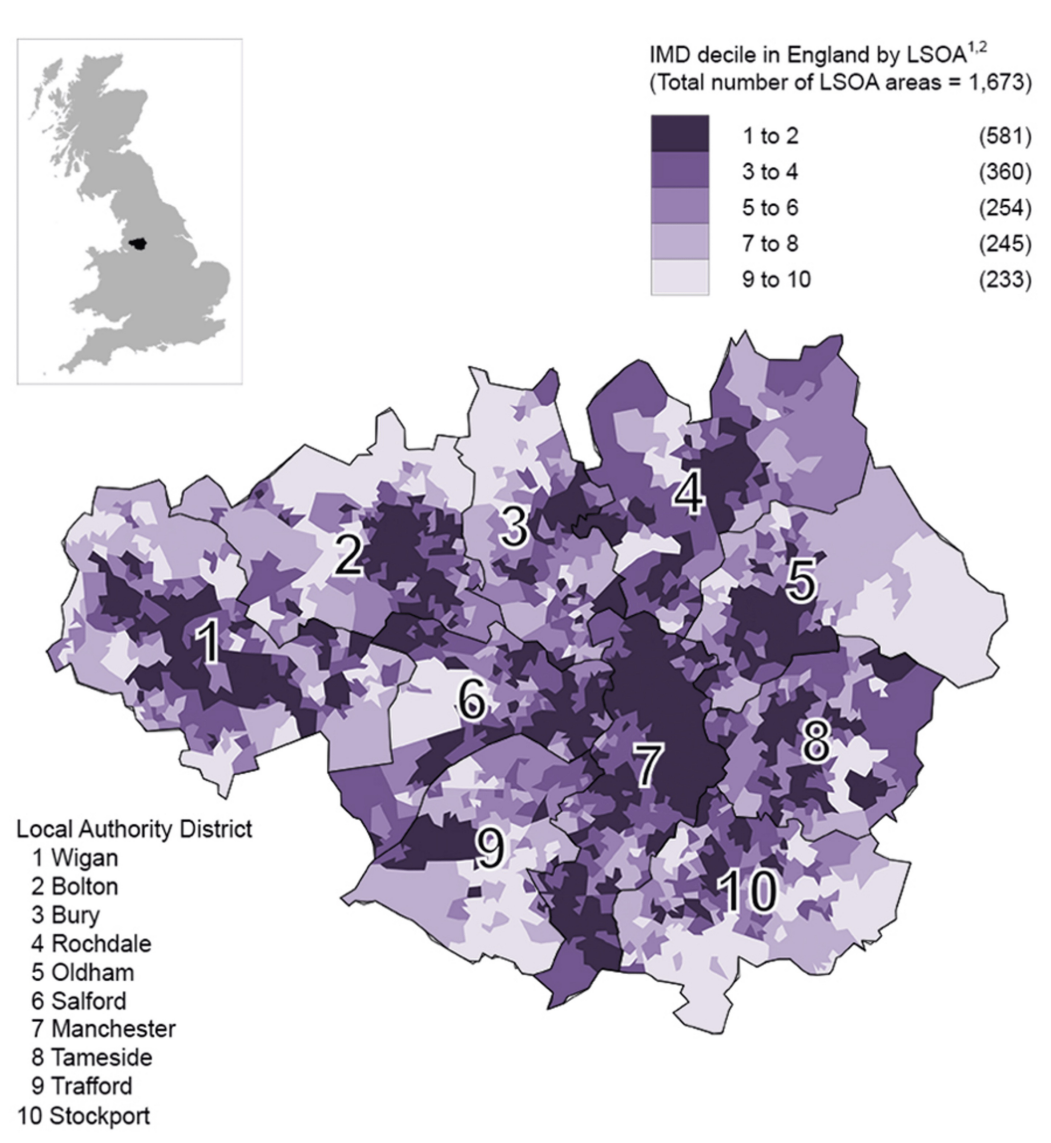
Displaying the distribution in Index of Multiple Deprivation in 2019 by LSOA across Greater Manchester LSOA: Lower-layer super output area Source: Department of Communities and Local Government (DCLG); Office for National Statistics licensed under the Open Government Licence v.3.0

**Figure 2:**
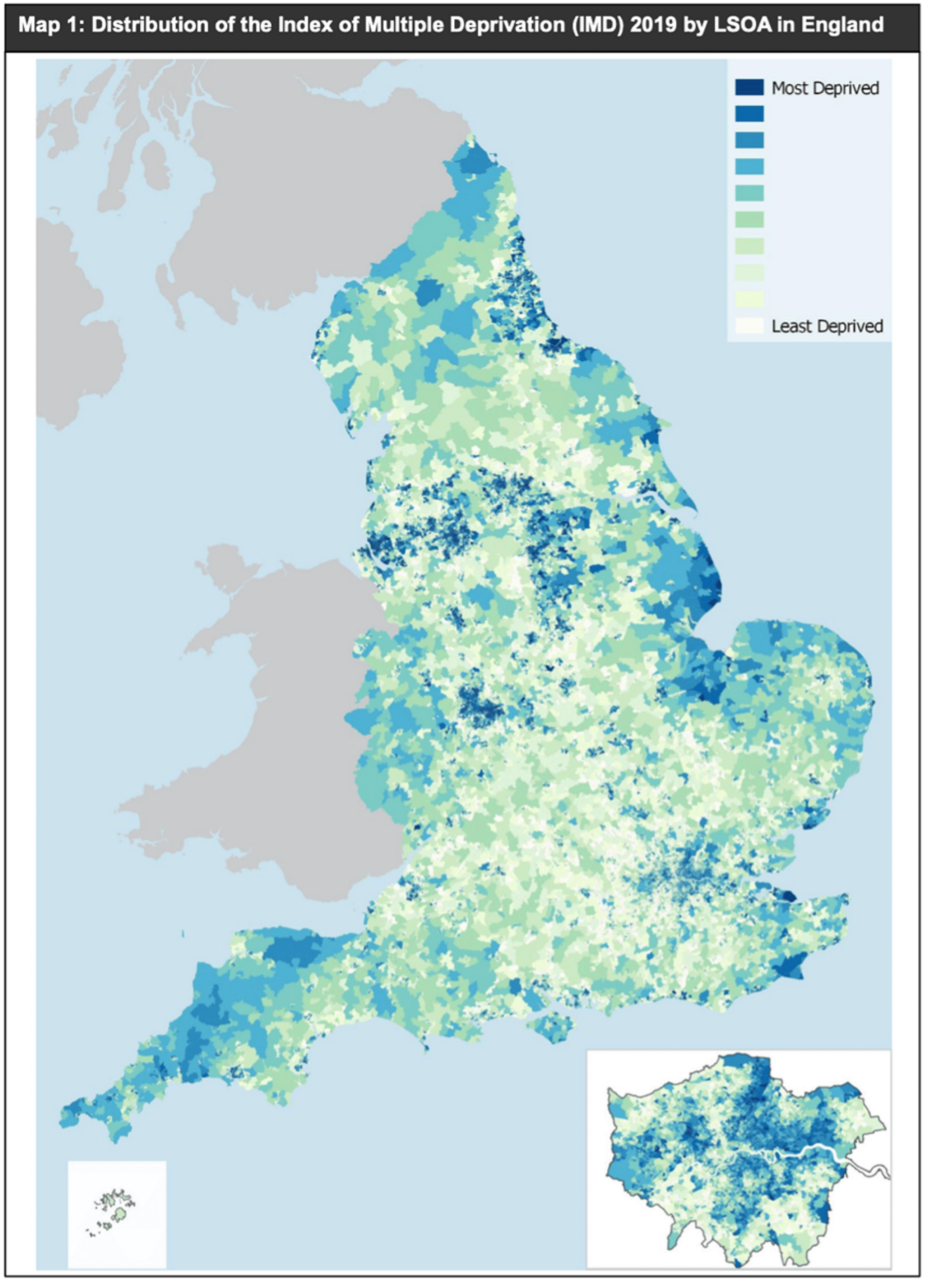
Distribution of the Index of Multiple Deprivation (IMD) 2019 by LSOA in England LSOA: Lower-layer super output area Source: © Ministry of Housing, Communities & Local Government, 2019

Nationally, Manchester ranks as the second most deprived local authority based on rank, the fifth most deprived based on score, and the fifth most deprived based on the proportion of LSOAs in the most deprived 10% nationally. For our analysis, we assigned each patient an IMD decile based on the LSOA of their registered home address at the time of hospital admission for hip fracture. This method enabled us to investigate the association between socioeconomic deprivation and the incidence of hip fractures, allowing us to control for socioeconomic variables and focus on the relative impact of deprivation on health outcomes. The distribution of IMD deciles based on LSOAs across Greater Manchester is shown in Figure 1, followed by Figure 2 illustrating the distribution across England.

## Results

The study cohort consisted of 585 patients. After excluding 15 overseas patients, 570 patients were included in the final analysis. The mean BMI of the cohort was 23.5, with the average age at the time of fracture being 81.1 years. Majority of the patients underwent surgical intervention on the same day as their presentation, with the mean time from presentation to surgery being 0.9 days. On average, patients were discharged from the orthopaedic ward 12 days post-presentation, with the overall average discharge time from the trust being 29 days.

IMD decile

**Figure 3:**
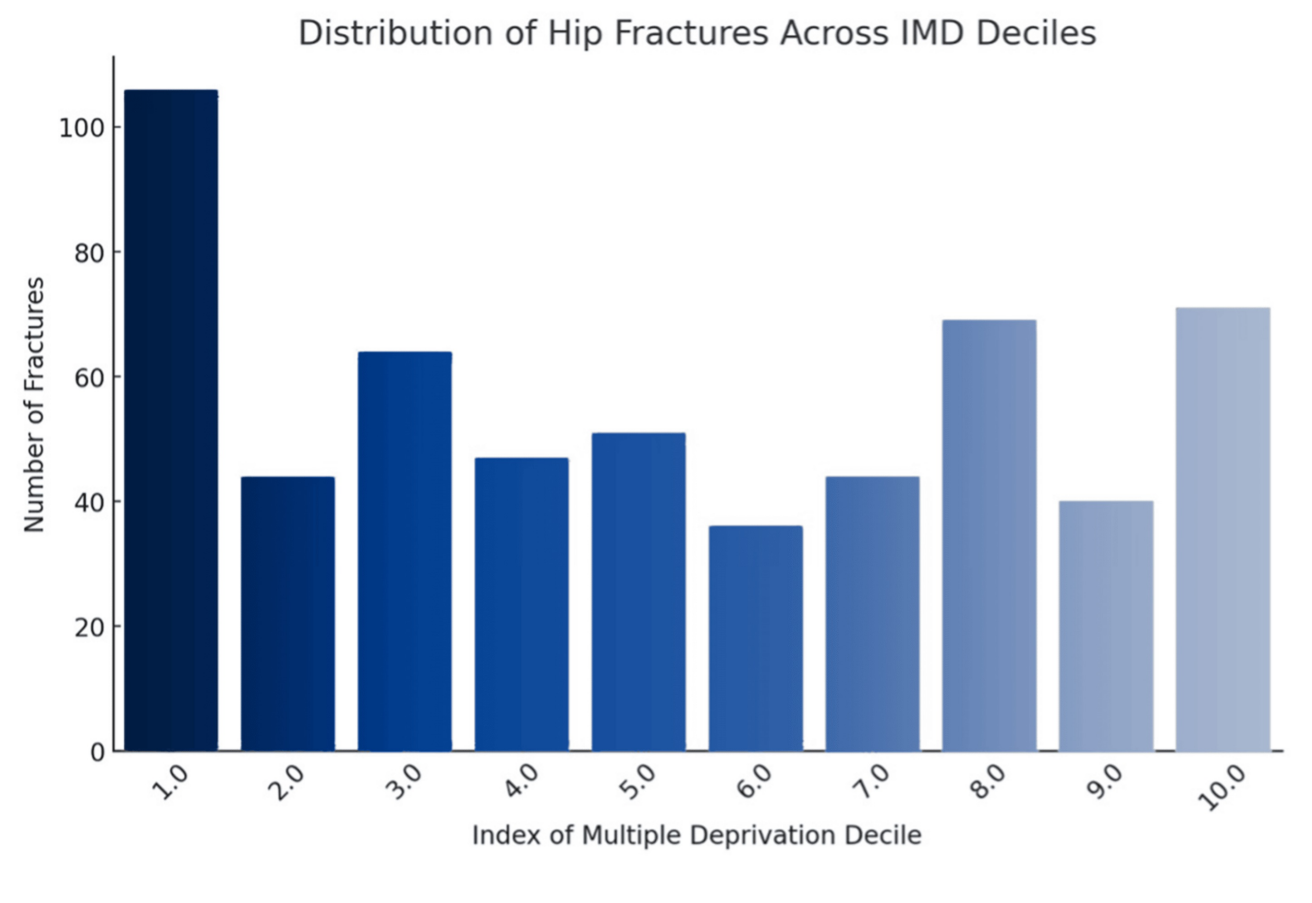
Frequency of hip fractures by Index of Multiple Deprivation (IMD) decile, highlighting socioeconomic patterns in fracture incidence

From the distribution in Figure 3, it is evident that the highest number of fractures occurs in the most deprived decile (Decile 1), with a notable incidence also observed in the least deprived decile (Decile 10).

Statistical analysis yielded a p-value of 0.013 (p < 0.05), demonstrating a significant correlation between socioeconomic status, as stratified by IMD deciles, and the incidence of hip fractures. These results indicate a non-uniform distribution of hip fractures across IMD deciles. Specifically, areas with higher deprivation (lower IMD deciles) exhibit a distinct pattern of fracture occurrence when compared to more affluent areas (higher IMD deciles), thereby reinforcing the notion that socioeconomic status is a significant determinant in the risk profile of hip fracture incidence.

IMD group and fracture type

**Figure 4:**
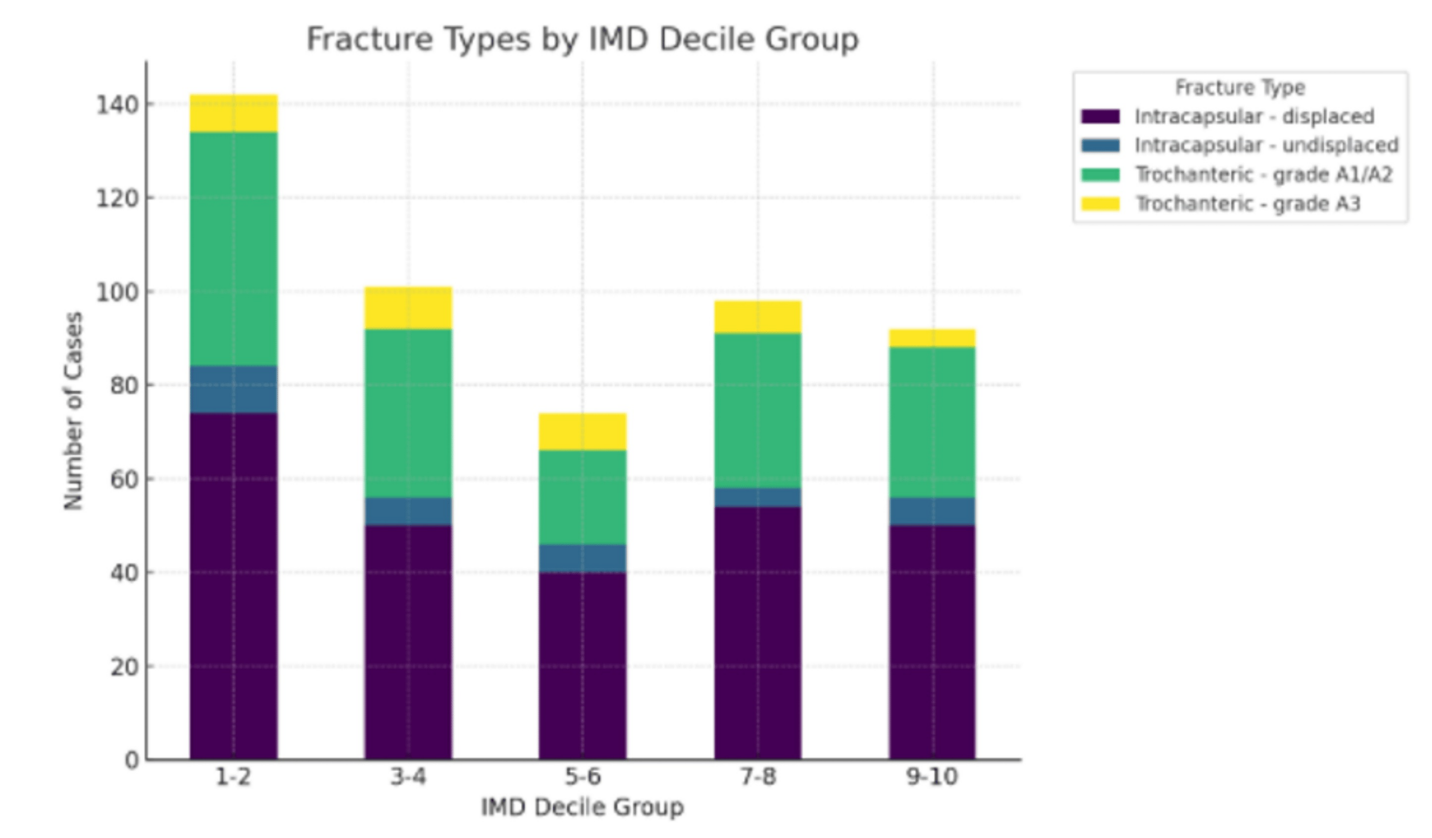
Distribution of the top 4 most frequent fracture types across Index of Multiple Deprivation (IMD) decile groups in pairs (1-2, 3-4, 5-6, 7-8, 9-10)

Figure 4 provides insight into the distribution of hip fracture types across different socioeconomic groups, as categorised by the IMD. The findings indicate a marked prevalence of intracapsular displaced fractures within the most deprived IMD groups (Deciles 1-2), with 98 cases reported. This type of fracture demonstrates a consistent decrease in occurrence as the IMD decile increases, suggesting a direct correlation between higher incidence rates and lower socioeconomic status. Similarly trochanteric fractures (grades A1/A2) are predominantly high in the lowest IMD group, with 69 cases reported, although their distribution across other IMD groups is more balanced.

The results of the Chi-square test yielded a p-value of 0.113 (p > 0.05). This indicates that there is no statistically significant difference in the distribution of IMD deciles across different fracture types. Consequently, there is insufficient evidence to conclude that the distribution of deprivation levels significantly varies by the type of hip fracture experienced. This implies that while socioeconomic status may affect the likelihood of sustaining a hip fracture, it does not significantly influence the specific type of fracture that occurs.

Patient residence pre- and post-discharge

The assessment of patient residence before and after admission revealed that majority of patients (500 out of 573) lived in their own homes or sheltered housing prior to admission. This number decreased to 356 after discharge, with a notable increase in transitions to institutional care settings, including rehabilitation units (78 patients), nursing care (47 patients), and residential care (43 patients). Mortality statistics showed 23 deaths at discharge, 15 post-discharge, and two pre-surgery, totaling 40 deaths.

A significant change in residence was observed for 168 patients following their fracture, indicating a considerable shift in living arrangements. Socioeconomic analysis using IMD deciles showed that patients from various socioeconomic backgrounds initially resided predominantly in their own homes or sheltered housing. Post-discharge, there was a marked increase in institutional care across all deciles, with notable transitions from Deciles 1 (most deprived) and 10 (least deprived) to rehabilitation units and residential care.

Statistical analysis employing the Chi-square test yielded a p-value of 0.329, indicating no statistically significant association between IMD decile and changes in residence post-hip fracture. This suggests that the variations observed in residence changes across IMD deciles may be attributable to random chance rather than systemic socioeconomic factors.

In conclusion, while the data indicates a significant shift in residence and care settings following fracture, there is insufficient evidence to establish a significant impact of socioeconomic status on these changes. Further research is needed to investigate other factors influencing postoperative care pathways and residence transitions.

BMI

**Figure 5:**
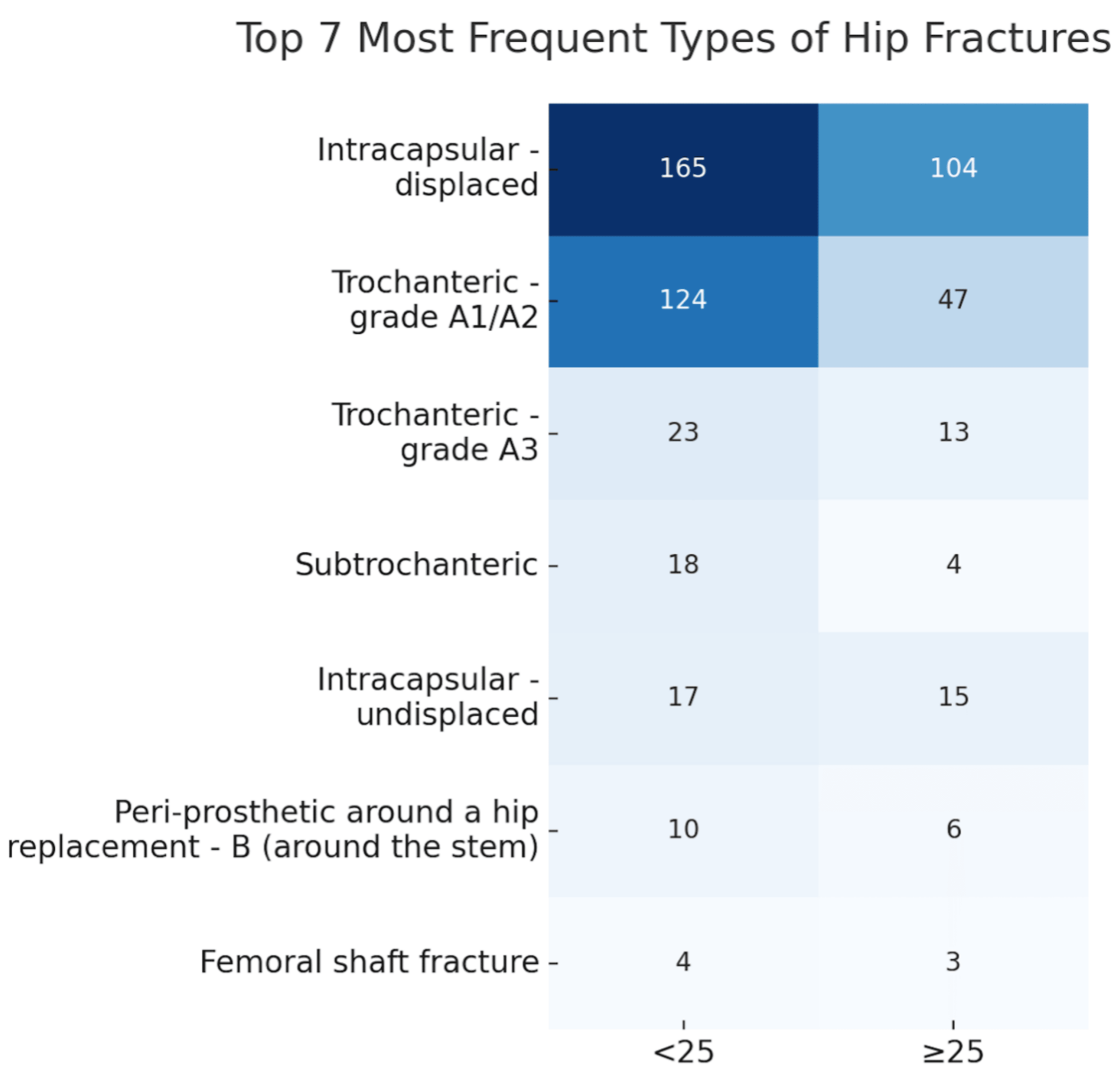
Illustrating the prevalence of the most common types of hip fractures categorised by individuals with a BMI of <25 and a BMI of ≧25 BMI: Body mass index The left column presents data for individuals with a BMI less than 25, considered as normal or underweight, whereas the right column presents data for individuals with a BMI of 25 or greater, typically classified as overweight or obese

To examine the relationship between BMI and hip fracture type, patients were categorised into two BMI groups: <25 and ≥25. A Chi-square test was conducted, yielding a p-value of 0.03 (p < 0.05). This result indicates a statistically significant association between BMI categories and fracture types, suggesting that BMI may influence the likelihood of sustaining particular types of hip fractures. The prevalence of the fracture type in each BMI group is shown in Figure 5.

The analysis reveals a nuanced relationship between BMI and fracture type. Specifically, a significant association was found between higher BMI (≥25) and the incidence of trochanteric-grade A1/A2 fractures, with a p-value of 0.030. This suggests that individuals with elevated BMI are at an increased risk for this specific fracture type. Conversely, no statistically significant associations were observed for intracapsular-displaced, subtrochanteric, or trochanteric-grade A3 fractures, as indicated by p-values exceeding the 0.05 threshold. These findings indicate the importance of considering BMI as a factor in the risk assessment for certain hip fractures types.

Age

**Figure 6:**
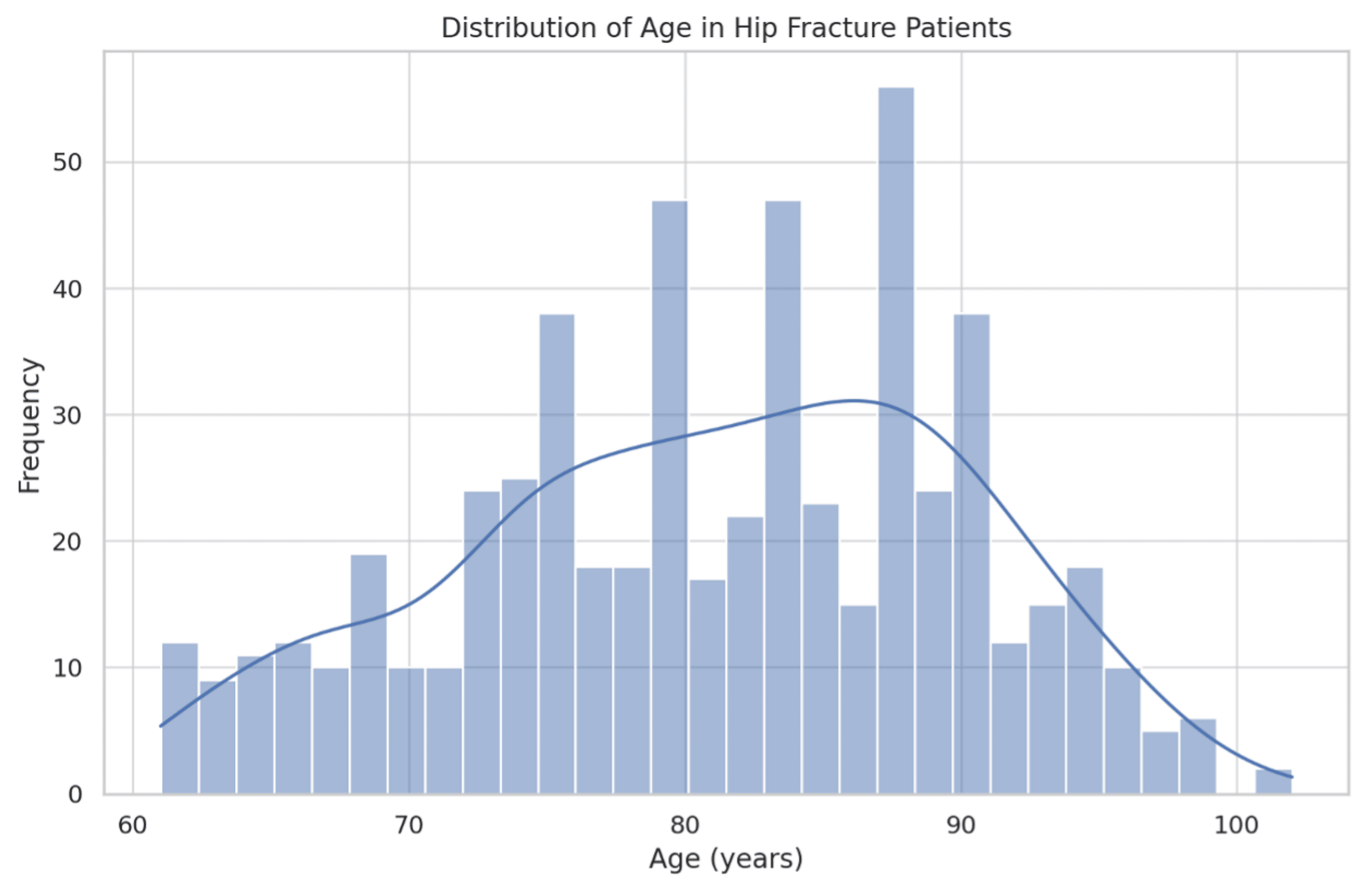
Age distribution of hip fracture patients, with frequency histogram and normal distribution curve

The study of hip fracture incidents revealed a modal age of 84 years, with a mean age of 81.1 years at the time of fracture. Figure 6 illustrates a skewed age distribution towards older individuals, aligning with clinical observations that the elderly are at increased risk due to diminished bone density and increased fall risk.

A detailed examination by fracture type, as presented in Table 2, showed variability in median, mean, and modal ages. Intra-articular distal femoral fractures and peri-prosthetic fractures occurred predominantly in individuals in their late 80s to early 90s. The exception in peri-prosthetic fractures was that for fractures distal to the stem in hip replacements (group C as in Table 2) occurring in those less than 80 years of age. In contrast, extra-articular distal femoral fractures and intracapsular-displaced fractures were more common among those in their early 70s to 80s.

**Table 2:** Median, mean, and mode ages by fracture type The alphabets correspond to a grouping of the injuries as in the National Hip Fracture Database (NHFD) [1]. Further description of the group is included in parenthesis following the alphabet

Fracture type	Median age	Mean age	Mode age
Distal femoral fracture-extra-articular	74.5	74.5	70
Distal femoral fracture-intra-articular	93.5	93.5	88
Femoral shaft fracture	82.0	82.7	74
Intracapsular-displaced	80.0	80.2	80
Intracapsular-undisplaced	82.5	81.4	68
Peri-prosthetic around a hip replacement-A (trochanteric)	93.0	93.0	90
Peri-prosthetic around a hip replacement-B (around the stem)	83.0	83.0	75
Peri-prosthetic around a hip replacement-C (distal to stem/cement)	76.0	76.0	76
Peri-prosthetic around a knee replacement-A (epicondyles)	90.0	90.0	87
Peri-prosthetic around a knee replacement-C (proximal to implant/cement)	83.0	82.0	66
Peri-prosthetic around previous fixation device	90.5	90.5	90
Peri-prosthetic between a THR and a TKR-D (inter prosthetic)	85.0	88.3	84
Subtrochanteric	83.0	81.7	63
Trochanteric-grade A1/A2	83.0	81.3	89
Trochanteric-grade A3	82.5	81.0	88

Statistical analysis did not reveal a significant association between age and fracture type, suggesting that age alone is not a reliable predictor of specific hip fracture types. These results imply that while age contributes to the overall incidence of hip fractures, other factors must be considered to fully understand the risk and distribution of different fracture types.

Gender 

Our demographic analysis of hip fractures has identified notable differences in age and fracture type between genders. For female patients, the mean age at which hip fractures occur is approximately 81.7 years. The median age is observed at 82 years, while the mode age, representing the most frequent occurrence, is 87 years, indicating a higher incidence of hip fractures in the older age group.

In contrast, male patients present a mean age of approximately 79.8 years for hip fractures. The median age for these fractures is slightly lower, at 80 years, and the mode age is observed at 76 years. This data suggests that hip fractures tend to occur at a younger age in males compared to females.

A t-test confirmed a statistically significant difference in age between genders, with a p-value of 0.016, highlighting a gender disparity in the age of fracture occurrence.

A Chi-square test of independence between gender and hip fracture type yielded a p-value of 0.0128 (p < 0.05), indicating a significant association. Intracapsular-displaced fractures, as well as trochanteric-grade A1/A2 and undisplaced intracapsular fractures, were significantly more common in females. Conversely, certain fracture types such as femoral shaft fractures and peri-prosthetic fractures around knee replacements were predominantly observed in females, while peri-prosthetic fractures around hip replacements-B (around the stem) were more frequent in males.

These findings suggest that gender not only affects the age at which hip fractures are most likely to occur but also influences the likelihood of sustaining specific fracture types, potentially reflecting underlying biological and lifestyle differences between sexes.

## Discussion

Our study, consistent with research from France [20] and England [21], establishes a clear link between higher hip fracture rates and areas of increased social deprivation, emphasising the need for further investigation into the underlying causes, such as healthcare access, nutritional deficiencies, environmental risks, and comorbid conditions. A 2019 study [22] highlighted the increased fall risk in socially deprived populations, suggesting that targeted interventions could help mitigate this issue.

Additionally, our study observed a trend, though not statistically significant, where many patients transitioned from independent living to assisted environments post-hip fracture, indicating that early interventions like home safety assessments and mobility training could prevent such transitions and reduce healthcare strain [23]. Expanding future study cohorts could provide the statistical power to confirm these measures' effectiveness, ultimately improving quality of life and reducing healthcare strain.

The findings also highlight the significant role of BMI in the risk of specific hip fractures, particularly trochanteric-grade A1/A2 fractures among individuals with a BMI of 25 or greater. This indicates the importance of targeted preventative care, including early detection, weight management, physical activity, and dietary interventions, to reduce fracture risks and improve patient outcomes. Furthermore, a statistically significant association between gender and hip fracture types was observed. These differences likely arise from biological and lifestyle factors unique to each sex. Targeted exercise programs to strengthen bone density and balance could reduce fracture risks in females, who are more prone to certain fractures [24]. Additionally, lifestyle changes, nutritional guidance, and tailored treatment protocols are needed to lower hip fracture incidence in both men and women [25].

The study was concentrated on the Greater Manchester region in England, which may limit the generalisability of the findings to other areas characterised by different socioeconomic contexts. The patterns observed in these regions might not be directly applicable to populations in other countries or regions with different healthcare systems, cultural norms, and socioeconomic structures. Moreover, while the study examined the impact of socioeconomic status on the incidence of hip fractures, it may not have comprehensively accounted for all potential confounding factors. Variables such as individual comorbidities, specific lifestyle behaviours, genetic predispositions, and variations in environmental risks might not have been fully controlled, which could influence the validity of the observed associations.

## Conclusions

In conclusion, our study highlights a significant correlation between socioeconomic status and hip fracture incidence, emphasising the critical role of social determinants in health outcomes. The disproportionate prevalence of hip fractures in socioeconomically deprived areas highlights the urgent need for targeted interventions that address both medical and social dimensions of fracture prevention. To reduce these disparities, it is essential to enhance healthcare access and quality in deprived communities, alongside intensified education on fracture prevention and healthy lifestyle choices. Healthcare policies must integrate strategies specifically targeting high-risk populations to address pervasive health disparities affecting socioeconomically disadvantaged groups. Such measures are critical for fostering a healthcare environment that supports improved outcomes for all patients, regardless of economic status.
